# The Use of Selected Bacteria and Yeasts to Control *Vibrio* spp. in Live Food

**DOI:** 10.3390/antibiotics8030095

**Published:** 2019-07-18

**Authors:** Javad Sahandi, Patrick Sorgeloos, Hui Xiao, Xianghong Wang, Zizhong Qi, Yanfen Zheng, Xuexi Tang

**Affiliations:** 1 College of Marine Life Science, Ocean University of China, Qingdao 266100, China; 2 Laboratory of Marine Ecology and Environmental Science, Qingdao National Laboratory of Marine Science and Technology, Qingdao 266071, China; 3 Lab of Aquaculture and Artemia Reference Center, Ghent University, 9000 Ghent, Belgium

**Keywords:** aquaculture, bacillus, lactobacillus, yeasts, resistance, *Vibrio*

## Abstract

*Vibrio* species are a significant causative of mass mortality in mariculture worldwide, which can quickly accumulate in live food and transmit into the larval gut. With restrictions on the use of antibiotics in aquaculture, finding a proper solution to reduce the risk of Vibriosis is vital. This study aimed to evaluate the susceptibility of *Vibrio harveyi*, *V. campbellii*, *V. anguillarum*, and *V. parahaemolyticus* to twenty-six bacterial and yeast strains and use the beneficial ones to enrich live food (Branchiopod, *Artemia franciscana*, rotifer, *Brachionus plicatilis* and copepod, *Tigriopus japonicus*). Thus, a modified disk diffusion method was applied. After a susceptibility assay, the bacteria and yeast beneficial in suppressing the *Vibrio* species were labeled by fluorescent stain and used to measure the accumulation potential in different live foods. Also, the beneficial bacteria and yeast were used to enrich live foods, and then the count of loaded *Vibrio* was estimated after 5, 10, 15, and 20 h by the serial dilution method. From the total bacteria and yeast strains that were used, *Candida parapsilosis*, *Pseudoalteromonas flavipulchra*, *Lactobacillus sakei*, *Bacillus natto*, and *B. amyloliquefaciens* inhibited all four *Vibrio* species. The results of microbial labeling showed that *L. sakei* in *Artemia*, *C. parapsilosis* in rotifers, and *V. harveyi* in copepods had the highest accumulation rate. The results of the estimation of loaded *Vibrio* in different live foods also showed that the use of beneficial bacteria and yeast each significantly reduced the count of *Vibrio*. Application of bacteria and yeast to suppress pathogenic *Vibrio* maybe a sustainable method for preventing this pathogen from harmfully invading aquaculture and may also aid in reducing the chances of antibiotic resistance in pathogenic *Vibrio*.

## 1. Introduction

Intensive larval fish production relies on the adequate production of live food. *Artemia*, rotifers, and copepods are widely used as live prey in marine aquaculture. *Artemia* is an easily accessible live food for finfish hatcheries. Rotifers, due to their small size, are a suitable food source for fish and crustacean larvae. Copepods are well known for their high level of unsaturated fatty acids, and their better balance between triglyceride and phospholipids. [1]. In spite of the mentioned usage of these three live foods and the other essential features of these organisms, the bacteria associated with these organisms are still the major concern. Sorgeloos et al. [2] reported that live foods are one of the primary carriers of bacteria that could cause larval diseases and mass mortality. With the increase in unqualified and contaminated aquaculture productions, such as finfish, crustaceans, and shellfish, bacterial diseases have emerged [3]. In mariculture hatcheries, *Vibrio* species are found everywhere. Vibriosis is a common disease caused by some species of *Vibrio*. These bacteria are the most common and severe pathogen in marine fish and shellfish aquaculture worldwide [4]. The production of fish larvae in commercial hatcheries still depends on the adequate supply of live prey, such as rotifers, *Artemia*, and copepods [5]. The remarkable inoculation of *Vibrio* species in live prey and the transmission of these pathogens into the larval digestive tract, or adhering to larval bodies’ surfaces, causes significant problems resulting in Vibriosis [2,6,7]. Vibriosis is a bacterial disease, so the use of antibiotics for reducing the mortality rate and curing the infected fish is unpreventable. The discovery of penicillin increased hopes for the control of infectious diseases, but soon bacterial resistance to antibiotics discomfited scientists [7,8]. Antibiotic resistance became a critical issue in public health and then extended to agriculture and aquaculture [7]. The microbial screen has started to become increasingly difficult. The spread of resistant bacteria has influenced coastal sea areas and aquaculture farms in a way that bacterial pathogens are now becoming resistant to most common antibiotics [9,10,11].

Recently, efforts have been made to develop strategies for microbial control in order to decrease the use of therapeutic chemicals and antibiotics [8,12]. The role of probiotics to limit and control environmental pathogens is particularly crucial for the future of aquaculture, particularly concerning the increasing number of antibiotic-resistant bacteria. Probiotics have been used to improve human health by decreasing the number of pathogenic bacteria in the digestive tract [12]. Then, with the achievement of new findings on probiotics and how they work, scientists began to use these beneficial bacteria to improve the growth performance of animals [13]. Probiotics have several benefits including efficient adherence to the intestinal epithelial cells to reduce or prevent the colonization of pathogens [12,14], competitive growth with pathogens [3], and also the production of metabolites to inhibit or kill pathogens [15,16]. Probiotics, defined as “live microorganisms which, when administered in adequate amounts, confer a health benefit on the host”, constitute a potential tool in the reduction of mortality in the rearing of aquatic organisms [12]. Generally, working on different beneficial bacteria and yeasts to find the best strain and define standard and simple methods for inhibition of specific pathogens, such as *Vibrio* spp., is still essential. The microbial susceptibility test is a research tool that could apply to determine the beneficial bacteria or yeasts, those which are helpful in the inhibition of particular pathogens. However, until now, something important has been missing, and that is the screen of the microbial accumulation rate in different live foods. 

The colonization procedure of pathogenic bacteria has been well explained by Olsson et al. [17]. For such studies, several techniques were employed, such as direct observation by electron microscopy or plating the organism’s homogenized body. Apart from laborious processes such as sampling and preparation of the sample for microscopy, none of these methods permit observation of organisms, nor do they guarantee that the observed bacteria are those of interest. Fluorescent labeling is a prospective method which can help in the following bacteria or yeast. Among the large variety of fluorescent stains, Hoechst 33342 (2′-(4-Ethoxyphenyl)-5-(4-methyl-1-piperazinyl)-2,5′-bi-1H-benzimidazole trihydrochloride) is a membrane-permeable and fluorescent DNA stain with low cytotoxicity that intercalates in the A–T regions of DNA, and which is used for labeling cell nuclei. This compound is used to prepare fluorescent-labeled cells to study their nuclei; however, in this study, it was used for bacterial labeling. This technique allows the observation of bacteria and yeast inside the live prey by fluorescence microscopy.

The use of live prey in larviculture is still a necessity as micro-sized formulated diets are not an adequate food for many fish and shellfish species, at least for the early larval stages. This restriction is due to the poor enzymatic activity in the larval digestive tract. Live prey, with their non-selective feeding system, are significant carriers of contaminated material to the larval digestive tract, which significantly impacts the microbiota of the larvae and, in many cases, is responsible for significant mortality. Different live prey have different feeding styles, and their feeding styles are considered in order to assess their potential for carrying bacteria and yeast [12,18]. This study was carried out to evaluate the microbial susceptibility of four *Vibrio* species (*Vibrio harveyi*, *V. campbellii*, *V. anguillarum*, and *V. parahaemolyticus*) to the different bacterial and yeast strains and to reduce the loading of *Vibrio* species in three different live foods including *Artemia franciscana*, *Brachionus plicatilis*, and *Tigriopus japonicus*. Also, we estimated the accumulation rate of beneficial bacteria and yeast in mentioned live foods using the fluorescence labeling method.

## 2. Results

Results of microbial susceptibility assay are presented in Table 1. From a total count of twenty-six bacterial and yeast strains, some were effective to stop *Vibrio* strains. The final results showed that *C. parapsilosis*, *P. flavipulchra*, *L. sakei*, *B. natto*, and *B. amyloliquefaciens* were successfully stopped the growth of all *Vibrio* species.

The results presented in Table 2 show the different potential of microbial accumulation rates in different live foods after exposure to bacteria and yeast (*p* < 0.05). The findings showed that in *Artemia* nauplii, the highest accumulation rate belonged to the group which was enriched with *L. sakei* (3.56 ± 0.31 × 10^5^ cell/individual) in a way that no individual cell was distinguishable after microscopy (Table 3, *p* < 0.05). The lowest count was observed in the group which was enriched with *C. parapsilosis*. In rotifers, the highest accumulation rate was recorded in those that were exposed to *C. parapsilosis*, and the lowest one was recorded in the group that was exposed to *B. natto*. In copepods, the highest accumulation rate belonged to *V. harveyi* (3.62 ± 0.26 × 10^5^ cell/individual) and then was observed in *L. sakei* (3.47 ± 0.25 × 10^5^ cell/individual) and *B. natto* (3.11 ± 0.13 × 10^5^ cell/individual) respectively (Table 2; *p* < 0.05).

**Figure 1:**
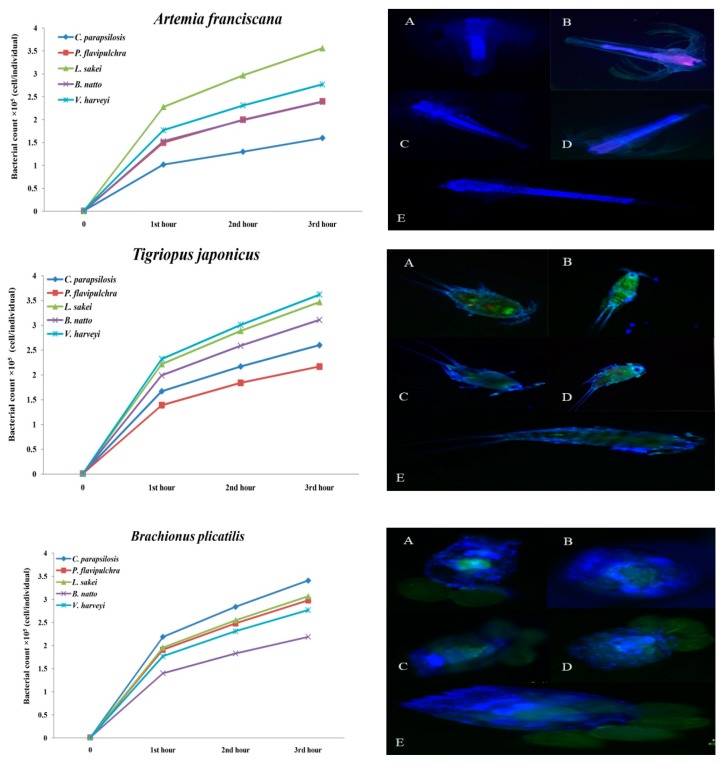
The results of the corrected total cell fluorescence of different microbial uptakes and a final comparison among different live foods recorded for three hours. The micrographs are presented to show the accumulation form of different bacteria and yeast in different live foods (*n* = 9; A: *Bacillus natto*, B: *Lactobacillus sakei*, C: *Pseudoalteromonas flavipulchra*, D: *Vibrio harveyi*, and E: *Candida parapsilosis*).

The results of the accumulation rate at various times are presented in Figure 1. In the group of *Artemia* nauplii after 3 h of enrichment, a significant increase in the accumulation rate of *L. sakei* (*r* = 0.96, *p* < 0.05) was observed and then in *V. harveyi*, *P*. *flavipulchra*, *C. parapsilosis*, and *B. natto* as well. In rotifers, the significant increase was observed in the treatment which was enriched with *C. parapsilosis* and then in *L. sakei*, *P*. *flavipulchra*, *V. harveyi*, and *B. natto*, respectively (*r* = 0.97, *p* < 0.05). The significant accumulation rates of *L. sakei*, *B. natto*, *C. parapsilosis*, and *P*. *flavipulchra* were recorded as well (*r* = 0.96, *p* < 0.05).

**Figure 2:**
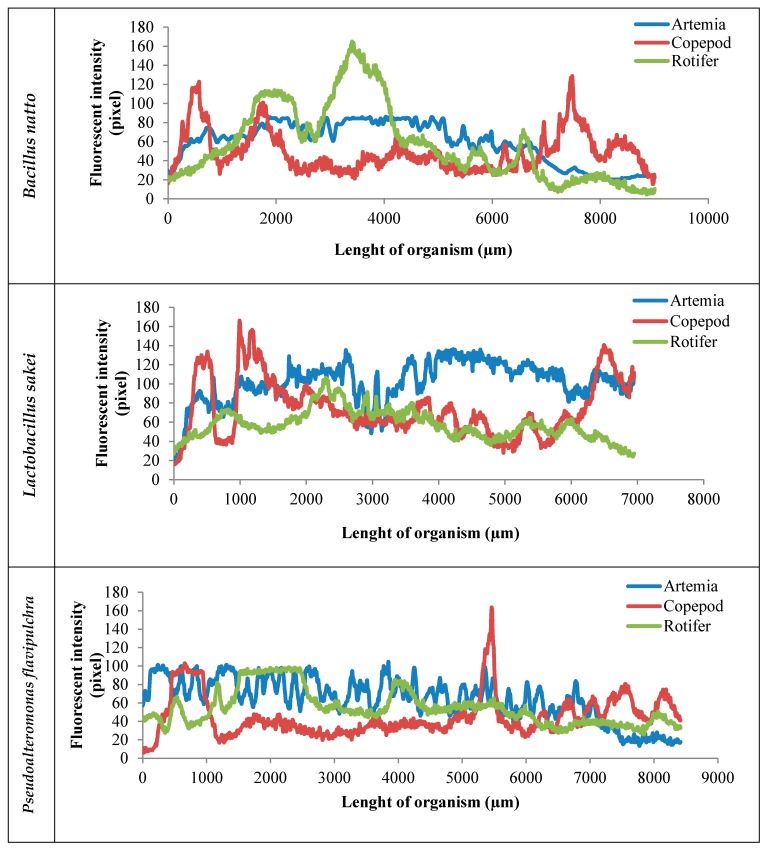

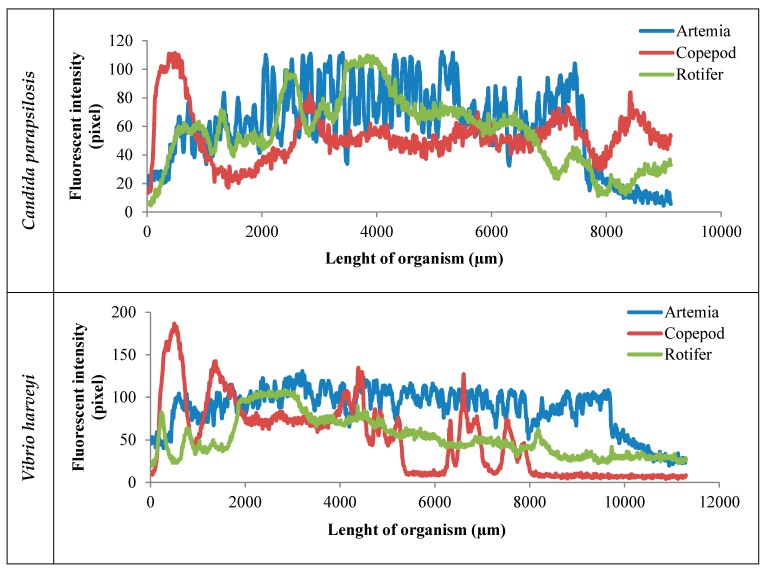
Representation of bacteria and yeast inoculation/adhesion rates alongside the different live foods’ (*Artemia franciscana*, *Tigriopus japonicus*, and *Brachionus plicatilis)* body after 3 h. Each graph refers to the count of labeled bacteria along the imaginary line that started from the head to tail of the organism, which was estimated by ImageJ software and the results are presented in the pixels (blue line refers to *Artemia*; red line refers to copepods; green line refers to rotifers; *n* = 15).

The fluorescent micrographs (Figure 2) show the different accumulation potentials of different bacterial and yeast strains in different live foods. The rotifer, as shown in Figure 1, showed significant potential for inoculation of bacteria and yeast. The highest concentration of bacteria and yeast in the group of *T. japonicus* was loaded on the body surface, especially between carapace fragments, antenna, tail, and legs. The highest accumulation rate in *Artemia* was concentrated in the digestive canal. Two bacteria, *B. natto* (A) and *P. flavipulchra* (C), besides their high ingested rate, also adhered well to the swimming appendages (Figure 1). 

The concentrations of *Vibrio* species after enrichment of different live foods with selected beneficial bacteria and yeast strains (*C. parapsilosis*, *P. flavipulchra*, *L. sakei*, *B. natto*) are presented in Table 3. The results documented that the use of beneficial bacteria significantly reduced the count of *Vibrio* species that were loaded in different live foods. In the *Artemia* group, *L. sakei* (1.19 ± 0.30 × 10^7^ CFU/g), *B. natto* (1.25 ± 0.21 × 10^7^ CFU/g), and *P. flavipulchra* (1.77 ± 0.23 × 10^7^ CFU/g) significantly reduced the count of *Vibrio* when compared with the control (10.16 ± 0.1 × 10^7^ CFU/g) over 20 h of enrichment. The results of rotifers also showed significant differences in treatments which were enriched with bacteria and yeasts when compared with the control in a way that *L. sakei* (0.38 ± 0.01 × 10^7^ CFU/g) significantly reduced the count of *Vibrio* (*p* < 0.05). In copepods, the lowest concentration of *Vibrio* was recorded in the treatment that was enriched with *P*. *flavipulchra* (2.13 ± 0.35 × 10^6^ CFU/g) and *L. sakei* (1.19 ± 0.3 × 10^7^ CFU/g).

## 3. Discussion

Finding the most effective bacterium or yeast to suppress the *Vibrio* species and estimating the accumulation rates of selected microorganisms in different live prey were the aims of this study. The suppression of all four *Vibrio* spp. was the criteria for choosing the bacteria and yeasts. Our results showed that from twenty-six strains of bacteria and yeasts that were applied, only four bacteria and one yeast including *C. parapsilosis*, *P. flavipulchra*, *L. sakei*, *B. natto*, and *B. amyloliquefaciens* inhibited all four *Vibrio* species (Table 2). The antimicrobial activity of the applied bacteria and yeast could be because of different reasons, including antagonistic properties or competition among strains. Previous studies, such as Chen et al. [19], Georgievaet al. [20], Younis et al. [16], and Yu et al. [3] all approved the antibiotic activity of these selected microorganisms against the different pathogenic *Vibrio*. By considering the genus and properties of each of these beneficial strains, there are various reasons for their successful inhibition. From the *Bacillus* strains, *B. natto* and *B. amyloliquefaciens* inhibited all four *Vibrio*. Likewise, Chen et al. [19] reported that *B. amyloliquefaciens* successfully stopped *V. anguillarum*, *V. campbellii*, *V. vulnificus*, *V. parahamolyticus*, *Edwardsiella tarda*, *Streptococcus* spp., and *B. cereus* growth. The extracellular enzymes that are secreted by *Bacillus* spp. can be causative of antimicrobial activity in which the secreted enzyme may put pressure on pathogenic bacteria and reduce their concentration [15,21], since this is a competition between two microorganisms, so the chance of forming resistance bacteria is close to zero.

Overall, of the yeast strains those were applied in this study, the majority of them inhibited *V. anguillarum* and *V. parahaemolyticus*. However, just *C. parapsilosis* inhibited all four *Vibrio* species. This strain is a member of the Saccharomycetaceae family and is an opportunistic marine pathogen [22], but there is no report to show this yeast harms finfish, in contrast, a beneficial effect was reported. Luna-Gonzalez et al. [13] reported that the use of *Pediococcus parvulus* and *C. parapsilosis* in every ten days of a feeding trial significantly increased the growth of *Oreochromis niloticus* and *Oreochromis* spp. Although, some species of the genus *Candida* were found in the alimentary canal of rainbow trout, it seems that they have a beneficial effect on growth and digestion [23]. Yeast may have antagonistic effects on entero-pathogenic bacteria [24].

*Lactobacillus* strains like the mentioned microorganisms (*Bacillus* and yeast) caused different ranges of inhibition when applied in susceptibility assay. Among used *Lactobacillus* strains, *L. sakei* was the best strain in inhibiting all four *Vibrio* strains. Also, this strain significantly reduced the count of *Vibrio* species in different live prey compared with other beneficial strains (Table 3). One of the factors that might affect the inhibition potential of *L. sakei* is the secretion of organic acids, which is different in concentration in various strains [20]. Soccol et al. [25] reported that *Lactobacillus* spp. can produce organic acids and bacteriocins, which would be considered in their application for antimicrobial purpose. Similar findings reported by Aras-Hisar et al. [26] that *L. sakei* Lb 706 inhibited *Listeria monocytogenes*. In other research, the inhibition of *Vibrio* with the application of different *Lactobacillus* strains was reported by Koga et al. [27], in which forty-one strains of *Lactobacillus* were tested for antagonistic activity against nine strains of *Vibrio*. The *R. mobilis* only inhibited *V. anguillarum* while *P. flavipulchra* successfully inhibited all four *Vibrio* species. This strain also significantly reduced the loaded bacteria in different live foods over 20 h of enrichment (Table 3). The antimicrobial activity of this strain against the *V. anguillarum* was reported by Yu et al. [28]. In another study, Jin et al. [29], reported the inhibitory activity of the same strain against *V. anguillarum*, *V. alignolyticus*, *V. campbellii*, *V. harveyi*, *V. mimicus*, *V. parahaemolyticus*, and *V. tubiashii*. Isanansetyo et al. [30] reported similar findings on the inhibitory activity of *Vibrio* spp. in which the *Pseudoalteromonas* strain S2V2, close to our strain, was applied. Isnansetyo et al. [30] reported that the strain S2V2 is releasing antibiotic components. Besides, the bacteria antagonistic effect of other strains of this genus against different *Vibrio* was approved by Richards et al. [31]. The operation of different strains of this genus in the production of antibiotics is different from the others; members of this genus can produce free or cell-bound antibiotics, extra- or intracellular [16].

**Figure 3:**
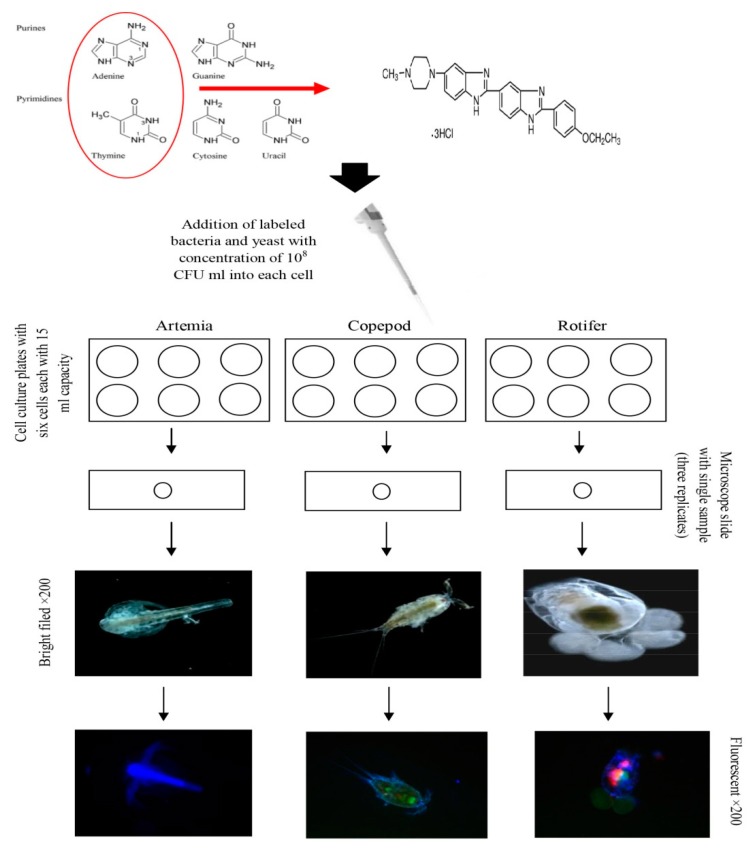
Schematic procedure of the fluorescent labeling of DNA and microscopy; this procedure was carried out for each one of the bacteria and yeast; the chemical structure of Bisbenzimide Hoechst 33342 (C_27_H_28_N_6_O), IUPAC name: 2′-(4-Ethoxyphenyl)-6-(4-methyl-1-piperazinyl)-1*H*,3′*H*-2,5′-bibenzimidazole.

What leads bacteria and yeast to be effective in the suppression of *Vibrio* spp. in live foods is the successful inoculation of these beneficial strains in host organisms [12]. The enrichment or bio-encapsulation of live foods is widely applied in aquaculture hatcheries for enhancing the nutritional value of live preys [18,20,25,32]. The results of the microbial accumulation rates in three different live foods, including *Artemia*, rotifers, and copepods showed a significant accumulation rate (*p* < 0.05). In the group *Artemia*, *L. sakei* (3.56 ± 0.31 × 10^5^ cell/individual) was the highest inoculated strain, and *C. parapsilosis* (1.60 ± 0.10 × 10^5^ cell/individual) was the lowest one (*p* < 0.05; Table 2). Similar to this finding, the best performance in the suppression of *Vibrio* spp. in different live foods was recorded in the treatment which was enriched with *L. sakei* as well. The accumulation speed and adhesion of bacteria and yeast alongside the nauplii showed a significant accumulation rate when compared with rotifers and copepods (Figure 2; Figure 3; *p* < 0.05). Besides, the results of enrichment of *Artemia* nauplii at different times showed that the use of *L. sakei* significantly suppressed the loading of *Vibrio* species (*p* < 0.05; Table 3). *Artemia* has primitive feeding characteristics which allow this organism to be a proper live food for carrying bacteria or yeast, whether a probiotic or pathogen. The study on the accumulation rates with the use of fluorescent microscopy was carried out to investigate the accumulation rates of different strains of beneficial bacteria in three different live preys. The high frequency of the graph alongside *Artemia* nauplii, shown in Figure 2, may refer to the microbial adhesion to the nauplius body surface. Ouwehand et al. [14] reported that some of the *Lactobacillus* strains, besides proper ingestion, also have a good potential for mucosal binding. Nevertheless, it is possible after a short-term incubation of live foods such as *Artemia*, rotifer or even copepod to replace opportunistic bacteria with useful ones and form a dominant population in live food in just 4–24 h [18]. In our study, a similar finding was observed; in rotifers and copepods the count of *Vibrio* during 5, 10, 15, and 20 h of enrichment was significantly reduced (*p* < 0.05). In contrast, in *Artemia nauplii* after 10 h of enrichment, the count of *Vibrio* started to increase. This could be because of the different metabolic speeds in different live foods. The adherence of bacteria to the epithelial cells of the host organism is an essential aspect for many mucosal pathogens and for their interactions [33]. This could be another reason that caused the suppression of *Vibrio* species. However, in the rotifer group, the highest accumulation rate belonged to *C. parapsilosis* (3.41 ± 0.12 × 10^5^ cell/individual), which after 20 h of enrichment, did not cause a proper suppression of *Vibrio* (6.53 ± 0.39 × 10^7^ CFU/g), but *L. sakei* (3.07 ± 0.11 × 10^5^ cell/individual) and *P. flavipulchra* (2.98 ± 0.49 × 10^5^ cell/individual) with the lowest accumulation rates showed the maximum effects on reducing the concentration of *Vibrio* species (*p* < 0.05). This means that success in the suppression of pathogenic bacteria may not be affected by the concentration of beneficial bacteria, which was already reported by Sahandi et al. [12].

One of the factors that might affect the inhibition potential of *L. sakei* is the secretion of organic acids, which is different in concentration in various strains. As it was reported by other researchers [32,34], yeast could be considered as a food source for the culturing of different live foods. The yeast *C. parapsilosis* also showed a rapid accumulation in comparison with those bacteria that were used. However, the results of the fluorescent microscopy showed the low frequency of fluorescently labeled bacteria alongside the rotifers’ body, which could be the result of the rotifers’ circular shape or the high ingested form of the labeled yeast in comparison with the other live foods (Figure3). The use of yeast as a food supply showed positive results when applied in the culturing of live prey, such as *Artemia* or rotifer. Patra and Mohamed [32] found that the enrichment of *A. franciscana* with *S. boulardii* after 24 h caused the accumulation of yeast at a level equivalent to 3.5 × 10^3^ CFU·g^−1^. The result of the copepods showed that *V. harveyi*, the candidate strain of *Vibrio* species labeled with fluorescent stains was highly accumulated in copepods (3.62 ± 0.26 × 10^5^ cell individual). This was carried out to show the accumulation potency of *Vibrio* spp. compared with beneficial bacteria. However, no significant differences were observed between *V. harveyi* and *L. sakei* (3.47 ± 0.25 × 10^5^ cell individual) and *B. natto* (3.11 ± 0.13 × 10^5^ cell individual) as well (*p* < 0.05). Also, *L. sakei* (1.19 ± 0.30 × 10^6^ CFU/g) and *B. natto* (2.59 ± 0.19 × 10^6^ CFU/g), with the same accumulation potential after 20 h of enrichment, significantly reduced the count of *Vibrio* spp. (*p* < 0.05; Figure 2; Table 3). This form of accumulation might be because of the living form of these bacteria, which is an attached living form [5,35,36]. Tang et al. [5] reported that copepods are suitable microbial hotspots, which means bacteria can load on the copepods’ body surface and multiply its colony. As shown in Figure 2, the frequency in the graph of *V. harveyi* alongside the body of *T. japonicus* was quite high. The fluorescent micrographs (Figure1) demonstrated that the outer body of the copepods accumulated with bacteria, especially among segments of the body. Tang et al. [37] reported that copepods in marine ecosystems provide a complex microhabitat, which is related to their body structure and large surface being potentially available for microbial colonization. The results of the effectiveness of the total used bacteria and yeast for susceptibility assay on different *Vibrio* species showed that *V. harveyi* and *V. campbellii* were the most resistant pathogens when compared with the other two species (*p* < 0.05). This study should be continued as a first step in the successful colonization of bacteria in live food to free pathogens. Using proper bacteria and yeast to reduce *Vibrio* loading in live food can be an important prospective purpose in marine larviculture. Further research should investigate the practical application of these strains in the production of live food.

## 4. Materials and Methods

### 4.1. Organisms and Culture Conditions

Four *Vibrio* species were obtained from the College of Marine Life Science, Ocean University of China (Qingdao-China). These bacteria including: *V. anguillarum* MCCO1 isolated from Codfish (*Gadus morhua*), *V. harveyi* MCCO1497, *V. parahaemolyticus* MCC56 isolated from Japanese Pufferfish (*Takifugu rubripes*), and *V. campbellii* MCCO1495 isolated from shrimp were the *Vibrio* species that were used in this study. Twenty-six different bacteria and yeast strains were obtained as well (Table 4).

The proper medium for each genus was obtained as follow:

1. Tryptic soy broth (TSB) for *Bacillus* strains. Composition (g/L): trypton—17, soya peptone —3, NaCl—5, K_2_HPO_4_—2.5, glucose—2.5. pH ~ 7.3; Sterilization—15 min at 121 °C.
2. Man, Rogosa, and Sharpe broth (MRS) for *Lactobacillus* strains. Composition (g/L): protose peptone—10, beef extract—10, yeast extract—5, dextrose—3, ammonium citrate—2, sodium acetate—5, magnesium sulfate—0.1, manganese sulfate—0.05, dipotassium phosphate—2. pH ~ 6.2 and Tween 80 1 mL^−1^; Sterilization—15 min at 121 °C.
3. Yeast extracts peptone dextrose broth (YPD) for yeast strains. Composition (g/L): yeast extract—10, peptone—20, dextrose—20. pH ~ 5.5; Sterilization—30 min at 115 °C.
4. E2216 broth. Composition (g/L) for Marine Bacterium strains and *Vibrio* species: Peptone—5, yeast extract—1, ferric phosphate—0.1. pH ~ 7.5–7.7; Sterilization—15 min at 121 °C.

Test tubes were filled with 10 mL^−1^ of different media and then were sterilized in an autoclave (Seisakusyo, Kagoshima, Japan). Then, the media were inoculated with selected bacteria and yeast strains under sterile condition. The bacteria and yeast were incubated in a rotary incubator (HDL, Model HZQ-F160, Shanghai, China) at 30 °C overnight. The cell-free culture of bacteria and yeast were prepared by centrifuging 5 mL^−1^ of each strain at 800× *g* for 10 min to separate the bacteria and yeast from the culture medium. The liquid supernatant was then discarded, and the pellet was suspended with sterilized saline solution (0.9% *w*/*v*). The microbial concentration was adjusted by spectrophotometer (UV 8000, Metash, Shanghai, China) at 610nm wavelength after Gomez-Gil et al. [47].

### 4.2. Microbial Susceptibility Assay

The susceptibility of four *Vibrio* species to various bacteria and yeast strains was determined using a modified disk diffusion method in which instead of diffusion disk, Oxford Penicillin cups were used. The concentration of 10^8^ CFU/mL of each bacteria and yeast strain was applied to inhibit the growth of each *Vibrio* species. The concentration of *Vibrio* was adjusted at 10^6–8^ CFU/mL as the highest possible concentration in marine environments. Each *Vibrio* species was spread on Muller–Hinton (MH) agar plates containing 2.5–3% NaCl separately. Then, they were left for 15 min [48] and five sterilized Penicillin cups were placed on each MH agar surface. The concentration of 10^8^ CFU/mL of different bacteria and yeast was added into each cup separately, each with three replicates, and then incubated at 30 °C for 24–48 h under aerobic condition. The diameter of the clear zone around each cup was measured with a caliper and recorded in mm.

### 4.3. Microbial Fluorescent Labeling

To investigate the accumulation rates of beneficial bacteria and yeast in *Artemia nauplii*, copepod, and rotifers, the beneficial bacteria and yeast including *P. flavipulchra*, *L. sakei*, *B. natto*, and *C. parapsilosis* were labeled by Bisbenzimide Hoechst 33342 (Sigma–Aldrich, Darmstadt, Germany) DNA marker. One of the *Vibrio* species (*V. harveyi*) also was labeled as well to make a clear comparison point between *Vibrio* and beneficial bacteria. The total amount of 1.5 mL^−1^ of bacteria and yeast containing 10–100 million cells was transferred into the sterilized centrifuge tubes separately. Then the bacterial solutions (culture medium and bacteria) were centrifuged at 800× *g* for 10 min. After centrifuging, the supernatant was released and the pellet at the base of each tube was re-suspended with 0.8–1 mL^−1^ of staining phosphate buffered saline (PBS) buffer. After, 5 µL of Hoechst 33342 staining solution (blue for the living cells, Technical Manual No. 0361) was added and incubated for 10–15 min at room temperature. The next solution was 5 µL of PI staining dye (red for the dead cells, Technical Manual No. 0361) which was added after the first incubation. After addition of PI, the suspension was mixed entirely and then put in an ice bath for 20–30 min. After final incubation for removing the rest of both stains, the bacterial solution (bacteria and PBS) was centrifuged again at 800× *g* for 10 min and wholly washed with the fresh PBS buffer. A total count of 50 individuals of *Artemia nauplii*, copepod, and rotifer were separately transferred into the cell culture plates with the capacity of 15 mL^−1^, each with three replicates. The labeled bacteria and yeast strains were added into each well with a concentration of 10^8^ CFU/mL and incubated for 3 h. During the three hours, different samples were randomly obtained in 1hour intervals. Three individuals of each live food were randomly captured from each replicate and then placed on a microscope slide (nine samples for each treatment). Excess water around the sample was dried with the use of the paper towel. The samples were then prepared, one after another, then the samples were placed on the stage of the fluorescence microscope in the upright position, and several micrographs were taken using a fluorescent light (Echo, San Diego, USA; 40×). Three different fluorescent lights, including green, red, and blue, were used to take the micrographs.

### 4.4. Live Food Culture

The cysts of *Artemia franciscana* were decapsulated and then hatched after Sorgeloos et al. [49]. The cysts were hatched in a 500 mL^−1^ conical flask, which was filled with seawater (31‰ salinity). The flask was placed inside a culture box with the proper aeration at 30 °C and permanent illumination. After 24 h, the hatched nauplii were separated from the cysts’ shell by positive phototaxis.

Harpacticoid Copepod, *Tigriopus japonicus* primary stock culture was obtained from Laboratory of Fanghong Mu, College of Marine Life Science, (OUC, Qingdao, China). The copepods’ culture was maintained in 50 mL^−1^ beakers filled with filtered seawater (31‰). The culture temperature was kept at 28 °C and a photoperiod of 12:12 h light: dark cycle was applied. The propensity of the culture salinity to increase due to the evaporation was adjusted by adding distilled water if needed to keep the salinity unchanged. Total water change was applied weekly with the use of plankton net with 25 µm mesh and the adult copepods and nauplii retained on the mesh were re-suspended into the beaker which was filled with newly filtered seawater. The copepods were fed on a combination of *A. tamarense*, the diatom *P. tricornutum* and beaker yeast, and *Saccharomyces cerevisiae* (Anqi^®^, Yichang, China) (2:2:1 ratio) every other day at 3 × 10^4^ cell/mL after Li et al. [34].

The stock culture of rotifers, *Brachionus plicatilis*, was obtained from the Institute of Oceanography, Qingdao Agricultural University, (Qingdao, China). The first stock has kept at 1 L^−1^ flasks containing 800 mL^−1^ of seawater with 31‰ salinity, 28 °C temperature and 12:12 h light: dark cycle with permanent aeration. The primary inoculum of rotifers culture was 50 individual/mL, with an initial 15–20 percent of ovigerous females. Rotifers were fed daily on microalgae (*Nannochloropsis oculata*) at a density of 10^4^ cell/mL [12].

### 4.5. Loading of Vibrio Speciesin Live Food

This experiment was carried out in a completely randomized design with four experimental treatments and the control with three replicates for each treatment. The proper count of each live food was separately transferred into glass containers which were filled with 400 mL^−1^ of filtered seawater (31‰) and kept at proper culture conditions as mentioned before. The concentration of 10^8^ CFU/mL of each bacterium and yeast (*P. flavipulchra*, *L. sakei*, *B. natto*, and *C. parapsilosis*) was obtained and added into the rearing water (400 mL^−1^) at the time zero and then after 5, 10, 15, and 20 h of microbial inoculation into rearing water and the count of *Vibrio* species was estimated after Mahios et al. [50]. In conducting this estimation, the live food (*Artemia* nauplii, rotifers, and copepod) samples from every single replicate were collected and then were weighed, homogenized, and serially diluted to 10^−7^ with sterilized saline solution (0.9%, *w*/*v*). Then, the amount of 100 µL of each solution was spread on TCBS agar plates. The plates were incubated at 28 °C overnight and the concentration was measured based on colony-forming units (CFUs). This experiment was repeated twice for each live food.

### 4.6. Statistical Analysis

Data including different *Vibrio* species responses to each bacteria and yeast strain in the susceptibility assay (mm) were entered in an Excel worksheet. The formula used to calculate the total percentage of effectiveness of bacteria and yeast strains was as follows: total percentage of effectiveness of single bacteria strain = quantity of useful strains to the single *Vibrio*/total strains used for test of each *Vibrio* × 100(%). The micrographs were taken by a fluorescence microscope (Echo, San Diego, USA) with 200× magnification. The count of cell-labeled bacteria and yeast cells was measured using ImageJ software (Java 1.6.0_20 (64-bit) version; http://imagej.nih.gov/ij) using the following formula: CTCF = integrated density − (area of selected cell × mean fluorescence of background readings). Data were analyzed as a completely randomized design and the regression of the bacteria count during the different times was estimated using SPSS software version 24. One-way ANOVA was performed. Tukey HSD multiple range tests were used to identify significant differences among different treatments (*p* < 0.05).

## 5. Conclusions

Increasing antibiotic-resistant bacteria is an important issue worldwide. The use of beneficial microorganisms, including bacteria and yeast, which is called probiotic, has been promoting over four decades. However, researchers found that the antagonistic activity of different strains of bacteria and yeast provides an excellent opportunity for preventing microbial infections in aquaculture farms. The primary step for using the probiotics is finding beneficial strains. In this study, the susceptibility of four pathogenic *Vibrio* spp. to different strains of bacteria and yeast was determined. The susceptibility test was carried out to find the best strain for the suppression of *Vibrio*. The results showed that five strains, including *C. parapsilosis*, *P. flavipulchra*, *L. sakei*, *B. natto*, and *B. amyloliquefaciens*, were successfully inhibited all four *Vibrio*. This result could be a great achievement, which is showing the potential of antagonistic activities of these strains when interacting with *Vibrio* species. Also, two strains, including *L. sakei* and *B. natto* besides inhibition latent, significantly reduced the count of *Vibrio* species in three different live preys over 20 h of enrichment. This suppression showed the high potential of these two strains in long-term inoculation in the digestive tract of these host organisms. Like this with increasing the use of these strains, besides reducing the treatment costs of mariculture farms, the growth and survival rate as it was reported before by several researchers would increase. However, still further studies need for well understanding the mechanism of microbial action against *Vibrio* species. 

## Figures and Tables

**Table 1:** The results of the susceptibility of *Vibrio* species to different strains of bacteria and yeast.

Yeast Strains	*V. anguillarum*	*V. harveyi*	*V. campbellii*	*V. parahaemolyticus*
*Candida pseudolambica*1401	++	+	−	+
*Candida parapsilosis*1019	+	++	++	+
*Pichia sydowiorum*3901	+++++	−	−	+
*Pichia sydowiorum*4901	+++	−	−	+
*Saturnisporasilvae*1501	++	−	−	+
*Torulaspora* spp. 82	++	−	−	+
*Pichia anomala* YF07b	++	−	−	++
*Pichia philogaea* K176	+++	−	−	+
*Candida* spp. 4201	+++	−	−	−
*Debaryomyces hanseii* K226	++++	−	−	−
**Marine Bacterium Strains**	***V. anguillarum***	***V. harveyi***	***V. campbellii***	***V. parahaemolyticus***
*Ruegeria mobilis* YJ3	++	−	−	−
*Pseudoalteromonas flaripulchra* JG1	+++	+++++	+++	++++
**Lactobacillus Strains**	***V. anguillarum***	***V. harveyi***	***V. campbellii***	***V. parahaemolyticus***
*Lactobacillus plantarum* JS201	−	−	−	−
*Lactobacillus plantarum* JS202	−	−	−	−
*Pediococcus acidlactici* JS203	−	−	−	−
*Lactobacillus sakei* JS204	++	+	++	++
*Lactobacillus bulgaricus* JS205	−	−	+	+
*Streptococcus thermophilus* JS206	−	−	−	−
**Bacillus Strains**	***V. anguillarum***	***V. harveyi***	***V. campbellii***	***V. parahaemolyticus***
*Bacillus laterosporus* JS01	++	−	−	+
*Bacillus coagulans* JS207	−	−	−	+
*Bacillus megaterium* JS02	−	+	+	−
*Bacillus natto* JS03	+++++	++	++++	+
*Bacillus mucilaginosus* JS04	−	++	++	−
*Bacillus amyloliquefaciens* JS05	+++	+++	++++	+
*Bacillus pumilis* JS06	−	+	+	−
*Bacillus subtilis* JS07	−	−	−	−

Note: − Resistance; + ≤10 mm; ++ <15 mm; +++ <20 mm; ++++ <25 mm; +++++ <30 mm.

**Table 2:** Corrected total cell fluorescence of three live food species (*Artemia*, Rotifer, and Copepod) exposure with labeled *Bacillus natto*, *Pseudoalteromonas flavipulchra*, *Lactobacillus sakei*, *Candida parapsilosis*, and *Vibrio harveyi* for three hours between organisms (*n* = 9; *p* < 0.05; Tukey HD).

Live Food	Microbial Strains (CTCF × 10^5^ cell/individual)				
	*C. parapsilosis*	*P. flavipulchra*	*L. sakei*	*B. natto*	*V. harveyi*
*A. franciscana*	1.6 ± 0.18 ^c^	2.40 ± 035 ^b,c^	3.56 ± 0.55 ^a^	2.39 ± 0.04 ^b,c^	2.77 ± 0.5 ^a,b^
*B. plicatilis*	3.41 ± 0.21 ^a^	2.98 ± 0.85 ^a,b^	3.07 ± 0.2 ^a,b^	2.19 ± 0.53 ^b^	277 ± 0.38 ^a,b^
*T. japonicus*	2.60 ± 0.36 ^a,b^	2.17 ± 0.11^c^	3.47 ± 0.44 ^b,c^	3.11 ± 0.24 ^a,b,c^	3.62 ± 4.66 ^a^

Means in the same row sharing the same superscript letter showed insignificant differences determined by Tukey’s test (*p* > 0.05). Data are expressed as mean ± S.E (*n* = 15).

**Table 3:** Mean values (±SD) of *Vibrio* species counts in three different live foods which were enriched with different bacteria and yeasts over 5, 10, 15, and 20 h.

Treatment	10^7^ CFU/g *Artemia* nauplii			
	5 h	10 h	15 h	20 h
Control	7 ± 0.1 ^c^	8.3 ± 0.2 ^c^	8.3 ± 0.4 ^c^	10.1 ± 0.1 ^d^
*C. parapsilosis*	2.9 ± 0.1 ^b^	3.7 ± 0.1 ^b^	2.7 ± 0.1 ^b,c^	4.9 ± 0.2 ^c^
*P. flavipulchra*	0.9 ± 0.2 ^a^	1.1 ± 0.2 ^a^	0.7 ± 0.3 ^a^	1.7 ± 0.2 ^b^
*L. sakei*	0.5 ± 0.1 ^a^	0.9 ^a^	1.4 ± 0.2 ^b^	1.1 ± 0.3 ^a^
*B. natto*	0.7 ± 0.1 ^a^	0.4 ± 0.1 ^a^	1.4 ± 0.2 ^b^	1.2 ± 0.2 ^a^
	10^7^ CFU/g rotifer			
Control	12.1 ± 0.3 ^d^	12.6 ± 0.3 ^d^	12.2 ± 0.5 ^d^	14 ± 0.3 ^d^
*C. parapsilosis*	8.0 ^c^	7.5 ± 0.2 ^c^	6.5 ± 0.2 ^c^	6.5 ± 0.3 ^c^
*P. flavipulchra*	1.6 ± 0.2 ^a^	1.1 ± 0.2 ^a^	0.4 ± 0.2 ^a^	0.2 ^a^
*L. sakei*	0.9 ± 0.1 ^a^	0.7 ^a^	0.4 ^a^	0.3 ^a^
*B. natto*	6.0 ± 0.2 ^b^	4.4 ± 0.5 ^b^	2.3 ± 0.4 ^b^	1.7 ± 0.1 ^b^
	10^6^ CFU/g copepod			
Control	10.6 ± 0.2 ^d^	10.8 ± 0.1 ^d^	11.3 ± 0.2 ^d^	13.1 ± 0.3 ^d^
*C. parapsilosis*	8.3 ± 0.2 ^c^	9.1 ± 0.1 ^c^	8.1 ± 0.2 ^c^	8.1 ± 0.2 ^c^
*P. flavipulchra*	3.3 ± 0.1 ^a^	1.1 ± 0.2 ^a^	0.7 ± 0.3 ^a^	2.1 ± 0.3 ^a^
*L. sakei*	5.3 ± 0.2 ^a,b^	3.7 ± 0.1 ^b^	2.5 ± 0.3 ^b^	1.1 ± 0.3 ^a^
*B. natto*	6.3 ± 0.5 ^b^	3.9 ± 0.2 ^b^	3.2 ± 0.1 ^b^	2.5 ± 0.1 ^b^

Different uppercase letters in the same column indicate significant differences (*p* < 0.05), the highest score express the lowest loading rate of *Vibrio*.

**Table 4:** The bacteria and yeast strains that were used for the susceptibility test.

Strain Code	Bacterial Strains	Reference	Strain Code	Yeast Strains	Reference
S4	^a^ *Bacillus subtilis*	[38]	82	^a^ *Torulasporaspp*	-
-	^b^ *Bacillus laterosporus*	[12]	1401	^a^ *Candida pseudolambica*	-
-	^a^ *Bacillus megaterium*	[39]	1501	^a^ *Saturnisporasilvae*	-
SRB-3	^a^ *Bacillus pumilus*	[40]	3901	^a^ *Pichia sydowiorum*	-
S18	^a^ *Bacillus amyloliquefaciens*	[41]	4201	^a^*Candida* spp.	[42]
-	^b^ *Bacillus natto*	-	4901	^a^ *Pichia sydowiorum*	-
- 1.19	^b^ *Bacillus mucilaginosus* ^c^ *Lactobacillus plantarum*	- [3]	6101 1019	^a^ *Rhodotorula mucilaginosa*	- -
				^a^ *Candida parapsilosis*	
-	^b^ *Bacillus coagulans*	[43]	K226	^a^ *Debaryomyces hansenii*	[44]
1.2696	^c^ *Lactobacillus acidlactici*	-	YF07b	^a^ *Pichia anomala*	-
1.6	^c^ *Lactobacillus sakei*	[45]	K176	^a^ *Pichia philogaea*	-
-	^d^ *Lactobacillus bulgaricus*	[46]	82		
- YJ3	^d^ *Streptococcus thermophilus* ^a^ *Ruegeria mobilis*	- -			
JG1	^a^ *Pseudoalteromonas flavipulchra*	-			

^a^ strains obtained from the College of Marine Life Science, OUC, Qingdao, China; ^b^ strains obtained from Wangfa Biology Co., Hebei, China; ^c^ strains obtained from the Department of Food Science and Engineering, OUC, Qingdao, China; ^d^ strains obtained from a commercial product (Beijing, China).
